# Metabolic responses to high protein diet in Korean elite bodybuilders with high-intensity resistance exercise

**DOI:** 10.1186/1550-2783-8-10

**Published:** 2011-07-04

**Authors:** Hyerang Kim, Saningun Lee, Ryowon Choue

**Affiliations:** 1 Department of Health Systems and Outcomes, Johns Hopkins University School of Nursing, 525 N, Wolfe Street Baltimore, MD 21205, USA; 2 Department of Medical Nutrition, Graduate School of East-West Medicine Science, Kyung Hee University, 1 Hoegi-Dong, Dongdaemun-Gu, 130-701, Seoul, Korea; 3 Research Institute of Medical Nutrition, Kyung Hee University, 1 Hoegi-Dong, Dongdaemun-Gu, 130-701, Seoul, Korea

## Abstract

**Background:**

High protein diet has been known to cause metabolic acidosis, which is manifested by increased urinary excretion of nitrogen and calcium. Bodybuilders habitually consumed excessive dietary protein over the amounts recommended for them to promote muscle mass accretion. This study investigated the metabolic response to high protein consumption in the elite bodybuilders.

**Methods:**

Eight elite Korean bodybuilders within the age from 18 to 25, mean age 21.5 ± 2.6. For data collection, anthropometry, blood and urinary analysis, and dietary assessment were conducted.

**Results:**

They consumed large amounts of protein (4.3 ± 1.2 g/kg BW/day) and calories (5,621.7 ± 1,354.7 kcal/day), as well as more than the recommended amounts of vitamins and minerals, including potassium and calcium. Serum creatinine (1.3 ± 0.1 mg/dl) and potassium (5.9 ± 0.8 mmol/L), and urinary urea nitrogen (24.7 ± 9.5 mg/dl) and creatinine (2.3 ± 0.7 mg/dl) were observed to be higher than the normal reference ranges. Urinary calcium (0.3 ± 0.1 mg/dl), and phosphorus (1.3 ± 0.4 mg/dl) were on the border of upper limit of the reference range and the urine pH was in normal range.

**Conclusions:**

Increased urinary excretion of urea nitrogen and creatinine might be due to the high rates of protein metabolism that follow high protein intake and muscle turnover. The obvious evidence of metabolic acidosis in response to high protein diet in the subjects with high potassium intake and intensive resistance exercise were not shown in this study results. However, this study implied that resistance exercise with adequate mineral supplementation, such as potassium and calcium, could reduce or offset the negative effects of protein-generated metabolic changes. This study provides preliminary information of metabolic response to high protein intake in bodybuilders who engaged in high-intensity resistance exercise. Further studies will be needed to determine the effects of the intensity of exercise and the level of mineral intakes, especially potassium and calcium, which have a role to maintain acid-base homeostasis, on protein metabolism in large population of bodybuilders.

## Background

Resistance exercise training is a principal anabolic stimulus for muscle protein synthesis and can result in hypertrophy of skeletal muscle [1-4]. Resistance training combined with a positive energy balance promotes muscle mass accretion synergistically [5]. Adequate protein intake is essential to optimize the rate of muscle protein synthesis sufficiently to attaining a positive net muscle protein balance [6]. It has been suggested that the consumption of 1.2-1.7 g protein/kg body weight (BW)/day or 25-30% of total calorie intake is recommended for bodybuilders to maintain muscle mass [7-9], yet a recent study of the bodybuilders showed intakes of protein of 34% of total calories [10].

If dietary protein and overall calorie intake are inadequate, body proteins will be broken down to meet the body's energy needs. On the contrary, overwhelming protein consumption significantly increases nitrogen and net acid excretion to maintain acid-base homeostasis and any failure of this mechanism can lead to metabolic acidosis [11-14]. Metabolic acidosis also promotes urinary calcium and phosphate excretion to counteract an increase in the circulating acid load produced by the catabolism of protein [15,16].

Metabolism of protein in the body is known to differ between exercising participants and non-exercising participants [17,18]. However, limited athlete-specific research on the effects of excessive dietary protein on metabolic homeostasis exists, even in groups of resistance exercisers. This study was undertaken to investigate the effect of high protein consumption on metabolic response in Korean elite bodybuilders participating in high-intensity resistance exercise training.

### Participants and methods

#### Participants

Eight Korean elite bodybuilders, who were defined by individuals who trained for competitions for over two years and had also won various national bodybuilding championships, were recruited. They were in the non-competition phase of training and exercised more than four times a week for over one and a half hours a day during this period of time. Exclusion criteria included those who took anabolic steroids or other drugs that can affect the metabolic acid-base balance. Participants with acute infectious disease, liver disease, kidney disease, or cardiovascular disease were also excluded.

#### Nutritional status

To determine dietary intake, three-day food records were used to assess the amount of ingested foods and number of daily meals (breakfast, lunch, dinner, and snacks). Athletes also recorded all of the supplements they were taking. Before starting, the participants were trained on how to record the total foods consumed in a daily record using common household measures by a skilled dietician. They were also instructed how to measure their portions using the utensils. The same dietician analyzed all food records by the Computer Aided Nutritional Analysis program version 3.0 (The Korean Nutrition Society, Korea).

#### Anthropometric evaluation

Body weight (kg), fat mass (kg, %), and lean body mass (kg) were determined by bioelectrical impedance analysis (BIA) (Inbody 3.0, Biospace, Korea) after fasting for 8 hours, wearing light clothing and no shoes. Body mass index (BMI, kg/m^2^) was calculated as body weight (kg) divided by squared height (m^2^).

#### Laboratory analysis

Blood samples were drawn after 12 hours of fasting to measure serum albumin, total protein, glutamate oxaloacetate transaminase (GOT), glutamate pyruvate transaminase (GPT), glucose, insulin, blood urea nitrogen (BUN), creatinine, calcium, phosphorus, sodium, and potassium. Glomerular filtration rate (GFR) was estimated using the methods of Daugirda [19].

Participants were required to collect their urine for a 24-hour period. They were instructed to urinate in the toilet and discard the first urine of the first morning of urine collection. Then they collected all urine for 24 hours and total volume, pH, osmolality and concentration of urinary urea nitrogen (UUN), creatinine, calcium, phosphorus, sodium, and potassium were determined.

All specimens except for serum insulin were sent to the laboratory and analyzed using standard methods with an automated chemistry analyzer (Hitachi, Tokyo, Japan). Serum insulin was measured by electrohemiluminescence immunoassay (Modular Analytics E-170, Roche diagnostics, USA).

#### Statistical analyses

Statistical analyses were performed using the SAS version 9.1. All numerical values are expressed as mean ± SD.

## Results

### Anthropometric characteristics

Anthropometric characteristics of the eight Korean elite bodybuilders are shown in Table 1.

**Table 1:** Mean age and anthropometric characteristics of the participants

Variables	Mean ± SD	Range
Age (yr)	21.5 ± 2.6	18.0~25.0
Height (cm)	175.5 ± 6.0	167.0~185.0
Weight (kg)	94.9 ± 12.9	79.3~117.4
BMI (kg/m^2^)	30.7 ± 2.6	27.4~34.3
LBM (kg)	74.4 ± 8.7	62.1~90.9
FM (kg)	16.4 ± 5.8	9.7~27.0
FM (%)	17.0 ± 4.4	12.3~25.6

BMI: Body mass index, LBM: lean body mass, FM: fat mass

### Daily nutrient intake

Participants consumed approximately 5,700 kcal/day: 4,948.7 ± 1,690.5 kcal from their diets and 673.1 ± 704.2 kcal from supplements, respectively (Table 2).

**Table 2:** Daily nutrient intake from diet and nutritional supplements

Nutrients	Diet	Supplements	Total
Energy (kcal)	4,948.7 ± 1690.5^1)^	673.1 ± 704.2	5,621.7 ± 1,354.7
Protein (g/d)	293.8 ± 137.0	112.2 ± 70.3	406.0 ± 101.1
Protein (g/kgBW)	3.1 ± 1.5	1.2 ± 0.8	4.3 ± 1.2
CHO:Pro:Fat (%Kcal)	37:24:39	14:66:20	34:30:36*
Ca (mg)	683.2 ± 389.5	1,494.4 ± 1,820.0	2,177.6 ± 1,588.5
P (mg)	2,704.3 ± 1116.9	564.3 ± 1262.4	3,268.6 ± 1,023.3
Na (mg)	4,081.1 ± 3337.9	823.8 ± 531.4	4,904.9 ± 3,168.9
K (mg)	5,043.6 ± 1998.8	909.3 ± 2,167.3	5,952.8 ± 2,135.9

^1) ^Mean ± SD

CHO:Pro:Fat: The ratio of carbohydrates, protein and fat of total calories consumed.

*34% of the total calories was derived from carbohydrates, with 95% from diet and 5% from supplements; 30% of the total calories was derived from protein, with 72% of protein being from diet and 28% from supplements; 36% of the total calories was derived from fat, including 93% from diet and 7% from supplements.

The proportion (%) of macronutrients to total calorie consumption was 34: 30:36 (carbohydrates: protein: fat). Energy acquired from protein and fat was relatively higher than the recommended amounts, while energy from carbohydrates was lower. The proportion of macronutrients from supplementary products was 14: 66: 20. About 28% of the total protein intake was obtained from commercial supplements, while carbohydrates and fat obtained from supplementary products made up 5% and 7%, respectively.

In addition, daily intakes of calcium and phosphate were 2,177.6 ± 1,588.5 mg and 3,268.6 ± 1,023.3 mg, respectively.

### Laboratory biochemical characteristics

The results of blood analyses are presented in Table 3. The values of albumin and total protein were within the normal ranges. The average value of GOT was 41.0 ± 19.3 IU/l, which was above the reference value. Half of the participants had a GOT value greater than 40 mg/dl, while a GPT level was within the normal reference value.

**Table 3:** Blood biochemistry values of the participants

Variables	Reference Value	Mean ± SD	Range
Albumin (g/dl)	3.1~5.2	4.7 ± 0.3	4.3~5.4
Total protein (g/dl)	5.8~8.1	7.7 ± 0.4	7.2~8.4
GOT (IU/L)	7.0~38.0	41.0 ± 19.3	26.0~84.0
GPT (IU/L)	4.0~43.0	37.8 ± 9.9	22.0~55.0
Glucose (mg/dl)	70~110	95.0 ± 7.6	85.0~108.0
Insulin (μU/ml)	2.6~24.9	2.9 ± 1.9	0.9~7.0
BUN (mg/dl)	6.0~23.0	19.9 ± 4.5	13.5~27.6
Creatinine (mg/dl)	0.5~1.3	1.3 ± 0.1	1.1~1.5
GFR (ml/min/1.73 m^2^)	80-120	78.3 ± 10.8	60.6-92.7
Ca (mg/dl)	8.2~10.8	9.2 ± 0.5	8.5~9.9
P (mg/dl)	2.5~5.5	3.7 ± 0.5	3.1~4.6
Na (mmol/L)	135~145	142.1 ± 1.4	141.0~145.0
K (mmol/L)	3.5~5.5	5.9 ± 0.8	5.1~7.2

GOT: Glutamate oxaloacetate transaminase; GPT: Glutamate pyruvate transaminase; BUN: Blood urea nitrogen, GFR: Glomerular filtration rate

Serum glucose (95.0 ± 7.6 mg/dl) and insulin (2.9 ± 1.9 μU/ml) levels were quite within the normal reference range. The BUN level was within the normal range (19.9 ± 4.5 mg/dl), and the serum creatinine level was on the upper limit of normal (1.3 ± 0.1 mg/dl). BUN and serum creatinine levels were elevated in 25% and 50% of the participants, respectively. The mean value of glomerualr filtration rate (GFR) was 112.8 ± 19.4 ml/min/1.73 m^2^, and it was elevated in 25% of participants.

Serum mineral levels, such as calcium, phosphate and sodium, were all within the acceptable reference values. The average level of serum potassium (5.9 ± 0.8 mmol/L, range of 5.1-7.2 mmol/L) was elevated above the normal range (3.5-5.5 mmol/L). Fifty percent of the participants had a value of potassium higher than the upper limit of the reference value.

The results of the urinalysis are presented in Table 4. Total 24-hour urine volume was 1,775.0 ± 489.2 ml/day, and the urinary pH was 6.3 ± 0.4. Urine osmolality was 810.8 ± 162.8 mosm./kg. Daily excretion of UUN was 24.7 ± 9.5 g/d, and all participants except one had a high value above the upper limits of normal. Urine creatinine was 2.3 ± 0.7 g/d and appeared to be higher than the reference range. Five (62.5%) participants had elevated urine creatinine. Urinary excretion of calcium was 0.3 ± 0.1 g/d, which was above the upper limits of normal, and 37.5% of participants had elevated value of urinary calcium. Urinary phosphate was 1.3 ± 0.4 g/d and was elevated in four participants. Urinary excretions of sodium and potassium were 91.8 ± 53.9 and 72.9 ± 33.7 mmol/d, respectively.

**Table 4:** Urine biochemistry values of the participants

Variables	Reference Value	Mean ± SD	Range
Urine volume (ml/d)	-	1,775.0 ± 489.2	1,100 - 2,500
Urine pH	4.8 - 7.5	6.3 ± 0.4	6.0 - 7.0
Osm. (m.osm/kg)	300 - 900	810.8 ± 162.8	519.0 - 1074.0
UUN (g/d)	6.5 - 13.0	24.7 ± 9.5	12.1 - 43.2
Creatinine (g/d)	1.0 - 1.5	2.3 ± 0.7	1.4 - 3.4
Ca (g/d)	0.1 - 0.3	0.3 ± 0.1	0.1 - 0.5
P (g/d)	0.4 - 1.3	1.3 ± 0.4	0.7 - 1.8
Na (mmol/d)	40 - 220	91.8 ± 53.9	28.0 - 199.0
K (mmol/d)	25 - 120	72.9 ± 33.7	25.0 - 134.0

UUN: Urine urea nitrogen; Osm.: Osmolality

## Discussion

### Diet characteristics

During the non-competition phase of training, one of the major goals of body builders is to increase muscle mass. Weight gain with a positive energy balance promotes an increase in muscle mass when combined with high-intensity resistance training [5]. Adequate protein intake is also required to provide the substrates for muscle accretion. Resistance exercise simultaneously increases both muscle protein synthesis and breakdown, but muscle protein synthesis overwhelms breakdown so that net muscle protein increases [20]. Therefore, in individuals engaging in an intense resistance training regimen, energy requirements and possibly protein requirements are increased. For these reasons, bodybuilders typically consume a high-protein diet in the non-competition phase of training.

There is as yet no definitive protein requirement for bodybuilders, however values in a wide range of 0.8 - 1.8 g/kg/day have been suggested [7,8,21]. The participants' average dietary protein intake in this study was 4.3 g/kg of BW/day, which was about 30% of their total caloric intake. The amount of protein was nearly five times higher than that recommended for the general healthy population (0.8 g/kg BW/day) [22]. It was also notably higher than any other recommendations of protein intake for bodybuilders, which have been suggested previously.

It is well known that a high-protein diet induces metabolic acidosis due to acidic residues of proteins. Metabolic acidosis induced by high dietary protein increases urinary acid excretion and also increases urinary calcium and phosphate levels, which may negatively influence bone and muscle protein metabolism. It is presumed that the participants who consumed excessive dietary protein (4.3 g/kg BW/day) in this study may have the risk of metabolic disturbance of acid-base homeostasis, based on the evidences from the previous study, which investigated the effect of high protein diet on metabolic acidosis.

Thus, this study suggested that it is important to determine the protein requirement for bodybuilders, because both over-intake of protein may induce unfavorable health outcomes.

### Urinary excretion of nitrogen in response to high protein diet

Protein-rich diets are acidogenic due to the release of excessive non-carbonic acids (e.g., sulfuric anions), which are produced by the metabolism of protein [11,13]. It is known that the activity of branched-chain ketoacid dehydrogenase is increased in response to a high protein intake [23]. This enzyme facilitates the oxidation and subsequent excretion of the increased amino group. Protein nitrogens are mainly excreted as urea nitrogen via the kidneys [24]. Urinary urea excretion has been shown to increase in response to an elevated dietary protein intake in resistance exercisers, suggesting that amino acid oxidation was increased [7]. On the other hand, the concentrations of urea in plasma and urine also increases during exercise and remains high for some time later, also in proportion to exercise intensity and duration [25].

In this study, the level of urea in plasma was within the normal range but elevated in 25% of the participants. The levels of UUN were twice as high as the recommended reference range. This result can provide an evidence to assume that elevated excretion of UUN might be due to the high rates of protein catabolism that follow high protein intake. Based on these results from increased UUN and creatinine, it is ascertained that dietary protein consumed by the high-intensity resistance exerciser might be mainly used as the substrates which is needed to release energy and/or to repair muscle mass during exercise.

### Urinary excretion of calcium in response to high protein diet

Urinary calcium excretion is ultimately affected by dietary calcium intake. However, high protein intake could not be completely excluded from influence on urinary calcium excretion. The amount of dietary protein as well as the amount of dietary calcium affects urinary calcium excretion [26]. It has been reported that the increases in urinary calcium excretion followed by high protein intake are similar to increases in urinary calcium excretion followed by high dietary calcium intake and independent of the level of dietary calcium [27]. A high-protein diet promotes renal calcium excretion by directly inhibiting renal tubular calcium re-absorption to maintain acid-base homeostasis [28-30]. In the previous interventional study, high protein diet significantly increased urinary calcium excretion in both human and animal model [14,31]. In the study of Wagner et al. [14], the urinary calcium excretion of the group received a high protein diet (2.0 g/kg BW/day) was almost two times higher than that of low protein diet group (0.5 g/kg BW/day).

However, although protein intakes (4.3 g/kg BW/day) in this study subjects were twice higher than the amount in Wagner et al.'s study, calcium excretion into urine was only on the border of upper limit of the reference range and the urine pH, which indicates the major evidence of metabolic acidosis, was still in normal range. It has been well-established that high protein intakes increase urinary calcium excretion in general population. However, there is limitation to fully explain the relationship between protein catabolism followed by high protein intake and urinary calcium excretion in the subjects with intensive exercise. It can be presumed that some factors, such as intensive exercise and other dietary factors, would play a role as buffer against increasing urinary calcium excretion in this subjects.

### The role of resistance exercise and dietary potassium on the preservation of nitrogen and calcium

Increased protein catabolism, accompanied by high-intensity exercise, may indicate bodybuilder have a higher rate of whole body protein turnover [32]. The participants in this study had high contents of muscle mass simultaneously with high UUN excretion. The plausible reason for increased UUN excretion might be the result from high rate of protein catabolism, using dietary protein as the substrate for muscle accretion. A high amount of dietary potassium also provides an anabolic stimulus for muscle synthesis and buffer against nitrogen excretion in urine [33]. Dietary potassium consumes H^+ ^and reduces both acid production and acid excretion [27]. Ceglia et al. [34], who studied the effects of a high-protein diet with supplementation of potassium bicarbonate on nitrogen excretion in healthy women, reported that UUN excretion reduced in the participants taking potassium supplements.

Nemoseck & Kern [35] recently investigated the effects of exercise on urinary calcium excretion, and they reported that urinary calcium excretion in participants who got intensive exercise was lower than those in the group that did not exercise. Dietary potassium also affects calcium metabolism and causes a positive calcium balance by directly or indirectly promoting renal calcium retention and inhibiting bone resorption [36-38].

In this study, participants were in the middle of intensive resistance training with multivitamins and mineral supplements. Multivitamins and mineral supplementation attributed to the high consumption of potassium along with other vitamins and minerals in all participants. The resistance exercise combined with the high dietary potassium intake might be possible to counterbalance the urinary nitrogen and calcium excretion induced by high intake of protein.

## Conclusions

This study was to investigate the metabolic response to high protein diet in elite bodybuilders with intensive resistance exercise. A large number of study results have previously shown the effect of high protein diet on metabolic acidosis in general population. However, the obvious evidence of metabolic acidosis in response to high protein diet in the subjects with high potassium intake and intensive resistance exercise were not shown in this study results. Several evidences in previous studies have shown that potassium intake and exercise could play a role as buffer against metabolic acidosis accompanied by high protein intake. Taking the view of metabolic responses to high protein diet, it can be presumed that excessive protein intake could lead negative health outcomes by metabolic changes. However, this study implied that resistance exercise with adequate mineral supplementation, such as potassium and calcium, could reduce or offset the negative effects of protein-generated metabolic changes.

This study was based on a cross-sectional design with a relatively small sample size, so it is limited when inferring causal links. Because of the study limitations, our results are mostly hypothesis-generated. Nevertheless, this study is constructive in providing preliminary information of metabolic responses to high protein intake in bodybuilders. Further studies would be required to determine the effects of the intensity of exercise and the level of mineral intakes, especially potassium and calcium, which have a role to maintain acid-base homeostasis, on protein metabolism in large population of bodybuilders. In addition, an experimental study to ascertain the safety and efficiency of protein intake in athlete group would be needed.

## Competing interests

HK, SIGL and RC declare that this study has no possible financial conflict of interest when submitting.

## Authors' contributions

HK and RC designed the study and were responsible for data analysis and interpretation. HK and SIGL contributed to screening and recruitment of participants and data collection. HK drafted the manuscript. RC supervised all procedure of this study and the manuscript. All authors read and approved the final manuscript.
